# Obituary

**Published:** 1850-07

**Authors:** 


### ARTICLE VII.

### OBITUARY.

Died, at his residence in Burlington, Iowa, April 26, 1850, of Pleuro-Pneumonia, LUTHER W. HICKOK, M. D., in the 38th year of his age.

Dr. Hickok was a native of East Bloomfield, N. Y. He added to professional acquirements of a high order, social qualities of an attractive kind, and his death will be mourned by his medical brethren as well as by his friends and former patients.

—On Saturday evening, 26th of May last, Dr. AMOS TWITCHELL, of Keene, N. H., in the 60th year of his age. He enjoyed a high reputation as a skilful practitioner.

—On the 9th of May last, M. GAY LUSSAC, the distinguished French chemist.

